# The effects of Ginger (*Zingiber officinale*) roots on the reproductive aspects in male Japanese Quails (*Coturnix coturnix japonica*)

**DOI:** 10.1186/s12917-023-03576-6

**Published:** 2023-02-03

**Authors:** Mostafa Galal Abdelfattah, Manal T. Hussein, Sohair M. M. Ragab, Nasser S. Abou Khalil, Abdelraheim H. Attaai

**Affiliations:** 1 Department of Poultry Production, Faculty of Agriculture, Assiut University, Assiut, Egypt; 2 Department of Cell and Tissues, Faculty of Veterinary Medicine, Assiut University, Assiut, Egypt; 3 Laboratory of Physiology, Department of Zoology, Faculty of Science, Assiut University, Assiut, Egypt; 4 Department of Medical Physiology, Faculty of Medicine, Assiut University, Assiut, Egypt; 5 Department of Anatomy and Embryology, Faculty of Veterinary Medicine, Assiut University, Assiut, Egypt; 6 Department of Anatomy and Histology, School of Veterinary Medicine, Badr University, New Nasser City, West of Assiut, Assiut, Egypt

**Keywords:** Ginger, Japanese male quails, Testis, Semen, Physiology, Histomorphometry

## Abstract

**Background:**

The Japanese quail is considered one of the most significant species in the poultry industry. However, the high male-to-female ratio results in the aggressive behavior of males. Dietary strategies that improve the properties of semen could reduce the number of males required to maintain optimal fertility and reduce aggressive behavior. Therefore, this study aims to provide insight into the possible improving efm fect of ginger roots on the reproductive aspects of Japanese male quails.

**Results:**

To achieve this objective, powder of Ginger roots was administrated to 2 groups of quails (10, and 15 g/Kg feed) from 7 days until 70 days of age. Some males were reared singly in cages (*n* = 40 for each group) to assess sperm quality and other males (*n* = 32 for each group) were raised with females to assess fertility and sperm-egg penetration. Additionally, biochemical tests and histological examination were also performed. When compared to the control group, dietary inclusion of Ginger at a dose of 15 g caused more improvement in ejaculate volume, sperm concentration, motility, viability and sperm-egg penetration. Whereas, the motility and fertility percentages of sperms were equipotent in both doses. Dose-dependent increases were found in the cloacal gland area and volume, as well as foam production and weight. Both doses resulted in a significant reduction in plasma total cholesterol along with an elevation cin plasma testosterone and lipid peroxides. The comparison between all groups concerning nitric oxide, catalase, superoxide dismutase, and total antioxidant capacity revealed the absence of significant difference. Morphologically, the diameter of the seminiferous tubules and the height of germinal epithelium significantly increased especially in the higher dose of Ginger.

**Conclusions:**

Ginger roots especially at a dose of 15 gm/kg feed was effective in improving male reproductive performance. These findings are of utmost importance in encouraging the addition of Ginger roots in ration formulation in male quails.

## Background

From the economic point of view, the Japanese quail (*Coturnix coturnix japonica*) is considered one of the most significant species in the poultry industry owing to its unique physiological features including rapid growth rate, effective reproductive potential, short life cycle, resistance to diseases, and early sexual maturity [1]. However, the aggressive behavior of males due to the high male-to-female ratio represents a major obstacle in front of breeding success and animal welfare [2]. The presence of numerous males creates a stressful social situation that emerges from the violent performance of cocks toward hens during sexual intercourse [3], and also increases the frequency of male-to-male confrontations aiming at social dominance [4]. This hostile environment has adverse sequelae on the health conditions of birds and fertility and hatchability of eggs [2]. Therefore, dietary strategies that improve the properties of reproductive potency could reduce the number of males required to maintain optimal fertility, and consequently diminish the aggressiveness and costs associated with male maintenance.

The disturbance in redox balance leads to a drop in testosterone production and degeneration of Sertoli cells ultimately resulting in a deterioration in semen traits culminating in low fertility [5, 6]. Male fertility indicators can be enhanced through the use of medical herbal with antioxidant properties by hampering oxidative stress, improving gonadal hormones leading to improvement in spermatogenesis and an increase in the reproductive efficiency of males [7].

One of the most promising natural candidates in this field is Ginger (*Zingiber officinale*). It enhanced the semen quality, fertility potential, and testicular histo-architecture in Japanese male quails and broiler breeders [8, 9]. The free radical quenching, androgenic and proliferative activities of Ginger satisfy the biological needs to achieve an effective reproductive function [7, 8, 10, 11]. Aqueous extract of Ginger roots enhanced spermatogenesis, the pituitary–gonadal reproductive axis, and semen biological properties in broiler breeders [12]. Supplementation of aged Cobb 500 breeder roosters with powder of Ginger roots could be a favorable dietary approach in attenuation of subfertility by improving various sperm attributes, seminal total antioxidant capacity, and fertility rate [13]. The increase in the cholesterol supply, testicular perfusion, secretion of gonadotropic hormones, expression of androgen receptors, and activity of cytoprotective agents, and the amelioration in redox disturbances and apoptosis are implicated in the reproductive boosting effects of Ginger [10, 14, 15]. It possesses an amazing phytochemical profile that enable it to improve the breeding potency. Zingerone up-regulates Ki-67 [16], a nuclear protein involved in proliferation and differentiation of spermatogonia. 6-Gingerol preserves the integrity of testicular cyto-architecture by activating the rate-limiting enzymes responsible for energization and stabilization of genomic material of germ cells [17]. Manganese, a trace element richly present in Ginger, plays a pivotal role in saving sperms from free radicals-mediated oxidative injury [18].

Much research was conducted to enhance the fertility of male birds using many compounds. Vitamin E alone [19, 20], selenium [21], and nano-selenium [22], combined vitamin-E selenium [23], or n-3 and n-6 fatty acid [20] were used to enhance the fertility of male Japanese quail. However, Ginger was also used as an extracted oil [8]. Therefore, the present study aims to investigate the effects of Ginger roots’ powder (as a raw cheap material), at two selected doses, on the fertility of male quail through investigation of semen characteristics, testosterone level, redox status, and testicular histomorphometric features in the hope of translating it to commercial use.

### Materials and Methods

## Ginger root powder

The Ginger roots were obtained from a local herb store in Assiut, Egypt. To keep freshness, Ginger roots were minced weekly and mixed with a commercial mash diet that was utilized within a week. The used doses were at the rate of 1) no Ginger in the control group, 2) 10 gm of powder of Ginger roots/Kg feed, and 3) 15 gm of powder of Ginger roots/Kg feed.

## Characterization of phytochemical constituents of Ginger roots

It was performed by Analytical Chemistry Unit (Department of Chemistry, Faculty of Science, Assuit University, Assuit, Egypt) using GC–MS (7890A-5975B).

## Birds and experimental design

Four hundred fifty healthy one-day-old Japanese quails were used in this experiment. A visual inspection, activity and behavioral observations were performed before each stage to ensure their health condition. This study was conducted at the Poultry Research Farm, Faculty of Agriculture, Assiut University, Assiut, Egypt. All procedures of the current study had been conducted following the University guidelines for the care of experimental animals. Ethical approval (aun/vet/4/0001) was obtained from the Committee of the Faculty of Veterinary Medicine, Assiut University, Egypt. The study is reported following ARRIVE guidelines. The stages of the experiment were as follows:

Stage 1: 450 mixed-gender Japanese quails (one day old), obtained from commercial quail stock at the Poultry Research Farm, were divided randomly into 3 groups (150 birds each) and were housed on the floor of brooding pens. They were fed the control ration for the 7^th^ day. Stage 2: birds were started to be fed on the 7^th^ day with the trial rations until the 70^th^ day of age, i.e. along the experiment. Stage 3A: sexual discrimination was made in the 4^th^ week, and a total of healthy (inclusion criteria) 120 male quails of similar weight (inclusion criteria), 40 for each experimental group, were housed in individual cages (20 × 20 × 25 cm) for semen collection. The experimental unit in this case is the single male bird. Stage 3B: of the remaining birds, healthy 64 female, and 32 male quails from each group were randomly subdivided into 8 replicates (8 females and 4 males in each replicate) and housed in the breeding cage (57 × 50 × 30 cm) to determine the fertility. The experimental unit in this case is the single cage. The birds were subjected to similar management and sanitary conditions throughout the experimental period.

## Environmental conditions and experimental diets

The temperature was gradually decreased from 35–37 °C at hatching to 20–23 °C at five weeks of age, and the relative humidity average was 60–65% up to the end experiment with adequate ventilation. The lighting program was 23 h of light:1 h of dark during the first 3 days, which was gradually decreased (one hour/week) to reach 16 h light: 8 h dark/day at 8 weeks of age. This regime lasted constantly till the end of the study (10 weeks of age). During the experimental period, feed and water were provided daily ad libitum, rations contained 24% crude protein (CP) and 2800 ME Kcal/kg (growing period) and 20% (CP) with 3000 kcal (ME)/Kg (production period). The detailed composition and calculated analysis of the experimental diets of growing and production periods are presented in Table (1).

**Table 1:** The composition and calculated analysis of basal diets of growing and production periods of Japanese quail

Ingredients %	Growing diet (%)	Production diet(%)
Yellow corn (8.5%)	56.44	63.02
Soybean meal (44%)	34.30	20.98
Corn gluten meal (62%)	6.30	8.34
Di-calcium phosphate	0.84	1.23
Limestone	1.18	5.51
Sodium Chloride (NaCl)	0.17	0.17
Sodium carbonate (Na2CO3)	0.16	0.15
L. Lysine	0.18	0.18
DL. Methionine	0.13	0.12
Vitamin and mineral Premix^a^	0.30	0.30
Total	100	100
**Calculated analysis**		
Crude protein%	24.00	20.00
ME Kcal/Kg feed	2800	3000
Linoleic acid%	1.38	1.47
Crude fiber%	3.72	2.96
C/P ratio	120.39	144.71
Calcium%	0.80	2.50
Available Phosphorus%	0.30	0.35
Sodium%	0.15	0.15
Chlorine%	0.14	0.14
Ash%	3.12	2.38
L. Lysine%	1.31	1.00
DL. Methionine%	0.50	0.45
Methionine + Cystin%	0.89	0.79

*ME * metabolic energy, *C/P ratio * calorie/protein ratio

^a^Each 3 kg of vitamin and minerals mixture contain: Vit. A, 10,000,000 IU; Vit. D3, 2,000,000 IU; Vit. E, 10,000 mg; Vit. K3, 1,000 mg; Vit. B1, 1,000 mg; Vit. B2, 5,000 mg; Vit. B6, 1,500 mg; Vit. B12, 10 mg; Niacin, 20,000 mg; Pantothenic acid, 10,000 mg; Folic acid, 1,000 mg; Biotin, 50 mg; Choline chloride, 500,000 mg; Copper, 4,000 mg; Iodine, 300 mg; Iron, 30,000 mg; Manganese, 60,000 mg; Zinc, 50,000 mg; Cobalt, 100 mg; and Selenium, 100 mg

## Semen evaluation

Semen collections were performed at 70 days of age (30 samples/group) from single males housed in the stage 3A cages. Before commencement of the semen collection, all the males were acclimatized to human handling and semen collection. Special care was taken to avoid any contamination during semen collection by clipping the feathers around the vent region and cleaning the proctodeal gland gently with tissue paper before semen collection. The semen collection from the male quails began by catching the male from cages. The male was gently restrained on the palm of the left hand. The wing and legs were held up and cloacal foam was collected separately in an aluminum paper by delicate squeezing the lateral wall of the foam gland with the thumb of the left hand and the forefinger of the right hand. The obtained foam was weighed directly following the semen collection. Semen was collected after massaging the lumber region of the bird 3–4 times smoothly and a gentle pressure was applied on either side of the vent using the thumb and forefinger. This technique was described by [24]. The semen was collected in graduated glass capillary tubes to quantify the volume and then diluted (1:9) semen: saline solution (NaCl; 0.9%, w/v) to evaluate the parameters of sperm motility. The sperm viability was assessed using one drop of blue staining eosin-nigrosin mixed with one drop of semen mixture on glass slides and evaluating 100 sperm cells at 400 × magnification [25]. The number of live cells (without color) and dead cells (pink color) was counted to calculate the sperm viability percentage (sperm viability % = live cells / total number of cells × 100). Live normal and abnormal sperm percentages were determined in a smear prepared for a live/dead sperm test. Sperm cell concentration was evaluated using the eosin stain. The spermatozoa were counted microscopically by the Neubauer Haemocytometer slide [26].

## Fertility test

Two thousand nine hundred forty-two eggs [Control 853 eggs + Ginger (10 g/Kg feed) 1238 eggs + Ginger (10 g/Kg feed) 851 eggs] were used to determine the fertility. The perfect fertile eggs laid for hatching from each treatment were collected daily and labeled to denote each treatment, for 5 consecutive days. Eggs were stored daily at 14 C° and 65% relative humidity in the egg holding room of the experimental hatchery for up to 5 days before being placed in the artificial incubator for each group. Eggs were transported to a hatchery machine and eggs were set in trays for incubation at 37.5C° temperature and 58–60% RH relative humidity for 1–14 days. On the 15th day of incubation, the eggs were transferred into pedigree boxes used for setting the eggs in different groups separated, and then placed in the hatchery. The temperature and relative humidity from 15-17 days of incubation maintained in the hatcher was 36.7 C° and 65%, respectively. At hatch time, all live and dead chicks were checked and counted while unhatched eggs were broken-out and examined microscopically to verify whether eggs were truly unfertilized or if they presented embryonic mortalities and know the total number of fertile eggs. The fertility percentage was calculated as ratios of the number of fertile eggs to the number of total eggs set in the incubator.

Fertility (%) = (number of fertile eggs/numbers of incubation eggs) × 100.

## Sperm-egg penetration assay

Sixty eggs (twenty eggs per group) were used for sperm penetration assay. The eggs were stored at 5ºC until they were analyzed. The assay was performed within two weeks after they were oviposited [27]. This test was performed on the level of different groups regardless the replicates. So, the twenty eggs were collected randomly from all replicates of the same group. The protocol included the removal of a portion around 1 cm^2^ of the perivitelline membrane. This portion of the perivitelline membrane was washed, straightened on a glass slide, fixed in 20% formalin, and finally stained with Schiff’s reagent (Sigma-Aldrich Company, USA). The number of sperm penetration holes in the whole germinal disc (SP holes/GD) was counted at the magnification of × 10 using a light microscope [28].

## Measurement of cloacal gland area and produced foam

One hundred and twenty males of the stage 3A (40 single males per group) were undergone to measure cloacal gland area using the digital caliber and foam production. The cloacal gland area (CAREA mm^2^) was used as an index of gland size. The lateral width (LW) and ventral-dorsal height (VDH) of the cloacal gland were measured to calculate the cloacal gland area by using a digital vernier caliper CAREA mm^2^ = (AG = LW × VDH) according to [29]. The cloacal gland volume (CVOL) was calculated according to the formula proposed by [30] (4/3 × 3.5414 × a × b^2^), where a = 0.5 × long axis and b = 0.5 × short axis).

The cloacal gland foam production (CFP) was quantified by subjective scaling of the amount of foam ejected, using a scale of 1 (no foam expressed) to 5 (maximum amount of foam expressed). The cloacal foam was collected individually by delicate, manual squeezing at the lateral wall of the foam gland with the thumb of the left hand and the forefinger of the right hand. The produced foam was collected on aluminum paper, which was put rapidly in airtight glass bottles to prevent evaporation of the foam and then weighed [31, 32].

## Blood sampling and biochemical parameters

At the end of the experiment (70 days of age), a representative number of male quails (*n* = 20) were randomly selected and slaughtered by the Islamic method which causes the least pain and suffering to the animals [33]. Blood samples were collected from the slaughtered birds into heparinized tubes and kept at 4 °C. The blood samples were immediately centrifuged at 3000 rpm for 15 min to separate plasma which was kept at –20 °C until used. After the bleeding stopped completely, the quails were de-feathered directly and eviscerated manually.

Biochemical parameters were estimated in 6 birds per group. Testosterone levels were determined by the ELISA technique using a testosterone enzyme immunoassay test kit (catalog number: BC-1115) according to the manufacturer's instructions (MYM Laboratory & Medical Supply, Inc., San Diego, USA). The intra-assay coefficient of variation is 7.4 while, the inter-assay coefficient of variation is 5.2 with a minimum detectable concentration is 0.05 ng/mL. Total cholesterol (TC), catalase (CAT), and total antioxidant capacity (TAC) were measured colorimetrically by commercially available kits obtained from Bio-diagnostic, Egypt (catalog number: CH1220, CA2517, and TA2513, respectively). Superoxide dismutase (SOD) activity was determined based on its ability to inhibit the autoxidation of epinephrine in an alkaline medium [34]. Nitric oxide (NO) was measured as nitrite concentration using the method of [35]. Lipid peroxides (LPO) were measured by thiobarbituric acid reaction according to the procedure of [36] using tetra-methoxy-propane as an external standard.

## Histological examination

For histological evaluation, specimens from the testes collected from a representative number of randomly selected quails (*n* = 5/group) were fixed in 10% buffered formalin solution in dibasic anhydrous sodium phosphate and monobasic acid phosphate (pH 7.0) for 48 h. Afterward, the fixed tissues were dehydrated with an ascending series of alcohols and cleared by immersing in xylene and then embedded in paraffin wax (Sigma Aldrich, USA) according to the standard methodology described before [37]. Transverse sections were cut at 5 µm thickness using a microtome (Richert Leica RM 2125, Germany) and mounted on glass slides. The testicular sections were deparaffinized with xylene and rehydrated through a decreasing gradient of ethanol followed by washing in distilled water. Then, the sections were stained with hematoxylin and eosin (H&E) for general histological examination and Crossmon’s trichrome for the identification of collagenous fibers. All the procedures were cited in [38].

The morphometric studies were performed on the light microscopic images of the quail^'^s tests sections of the control and Ginger groups using ImageJ software. The measurements were carried out on five randomly selected sections per five birds of each group.

## Statistical analysis

Data were presented as mean ± standard error (SE). The data obtained from the experiment were subjected to analyses of variance using the One-way ANOVA procedure of SAS 9.2 software statistical programs. The differences among treatment means were tested using Duncan’s multiple range test. Differences of *P* < 0.05 were considered to be statistically different.

## Results

### Characterization of phytochemical constituents of Ginger roots

The GC–MS revealed that cis-6-shogaol is the most prevalent compound in the Ginger roots (Table 2). Manganese level is 32.36 mg/kg.

**Table 2:** The phytochemical constituents of Ginger roots

Compound	Retention time (min)	% of total	Matching factor	Molecular weight
Cis-6-shogaol	16.08	25.303	38	276
Paradol	9.931	5.562	97	194
Gingerol	16.855	4.982	95	294
(1S,2S,3S,6S)-3-hydroxy-2-(Beta-hydroxymethyl)-1,7,7-trimethyl Bicyclo(4.4.0)Decane	18.715	3.154	38	240
Zingiberene	9.307	1.008	78	204
Zingerone	14.215	0.016	50	194

## Semen characteristics

The supplementation of Ginger led to improvement in most measured parameters of semen and fertility rate (Table 3). For instance, there was a significant increase in semen quality including ejaculate volume and sperm concentration. Moreover, sperm viability percentage, live/dead sperm percentage, live normal sperm percentage, and motility percentage were also improved. Furthermore, the abnormal sperm percentages were significantly lower in Ginger-fed Japanese quails when compared with the control group. Besides, the sperm penetration count in the Ginger-fed groups was significantly higher than its corresponding value in the control group. Consequently, the fertility rate in the Ginger groups was significantly higher than the control group.

**Table 3:** Effect of dietary supplementation of Ginger roots to Japanese male quails on semen characteristics, cloacal gland foam indices and sperm-egg penetration assay at 10 weeks of age

Group Parameter	**Control**	**Ginger (10 g/Kg feed)**	**Ginger (15 g/Kg feed)**	**Pooled SEM**	***P* value**
Ejaculate volume (μL)	5.47 ± 0.52^c^	13.43 ± 0.48^b^	16.40 ± 0.87^a^	0.599	0.000
Motility (%)	70.00 ± 1.17^b^	78.37 ± 1.38^a^	80.00 ± 0.81^a^	1.181	0.000
Sperm concentration (× 10^6^/mm^3^)	474. ± 10.44^c^	566.87 ± 21.89^b^	712.60 ± 15.33^a^	12.93	0.000
Sperm viability (%)	81.88 ± 1.00^c^	86.76 ± 0.78^b^	89.86 ± 0.78^a^	0.871	0.000
Live normal sperm (%)	68.38 ± 1.35^c^	76.51 ± 1.11^b^	81.9 ± 0.78^a^	1.28	0.000
Live abnormal sperm (%)	13.5 ± 0.45^a^	10.24 ± 0.41^b^	7.95 ± 0.59^c^	0.517	0.000
SP holes/GD	217.15 ± 3.69^c^	240.65 ± 3.95^b^	258.90 ± 3.86^a^	3.73	0.000
Fertility (%)	80.15 ± 2.13^b^	90.27 ± 0.95^a^	92.27 ± 0.85^a^	1.24	0.000
CAREA (mm^2^)	286.04 ± 4.16^c^	325.00 ± 4.97^b^	342.20 ± 6.90^a^	5.167	0.000
CVOL (mm^3^)	2736 ± 76.30^c^	3287.97 ± 76.80^b^	3594.33 ± 99.19^a^	79.70	0.000
CFP	2.88 ± 0.20^c^	4.24 ± 0.10^b^	4.66 ± 0.08^a^	0.138	0.000
Foam weight (mg/bird)	34.52 ± 4.27^b^	37.46 ± 2.74^b^	55.24 ± 0.08^a^	4.59	0.004

*SP holes/GD * number of sperm penetration holes counted in germinal disc, *CAREA* Cloacal gland area, *CVOL* cloacal gland volume, *CFP* cloacal gland foam production

Results are expressed as the mean ± SE of 30 Japanese male quails per group

^a,b,c^ Means in the same row with different superscripts are significantly different at p < 0.05 (One-way ANOVA followed by Duncan’s multiple range test)

## Cloacal gland index and different foam variables

The mean values of CAREA, CVOL, CFP, and foam weight in different groups of Japanese quails were presented in Table 3. Mean CAREA values in Ginger-fed groups were significantly higher than the control. The mean values of the foam gland and foam production measurements (CVOL, CFP, and foam weight) in Japanese quails supplemented with Ginger roots at a dose of 15 g/kg feed were significantly greater than those in the low Ginger and control groups.

## Biochemical findings

Table 4 depicted the changes in plasma testosterone and redox parameters in Japanese quails following the dietary inclusion of Ginger roots. The low and high Ginger groups were characterized by a significant increase in plasma testosterone and a significant decrease in plasma TC compared to the control group. Moreover, LPO levels in quails supplemented with Ginger roots were significantly higher than in the control group. However, there were no significant differences between all groups regarding plasma CAT, SOD, NO, and TAC.

**Table 4:** Effects of different doses of Ginger roots on the biochemical parameters in 70 days-old Japanese male quails

Group Parameter	Control	Ginger (10 g/Kg feed)	Ginger (15 g/Kg feed)	Pooled SEM	*P* value
Testosterone level (ng/ml)	1.15 ± 0.05^b^	1.86 ± 0.13^a^	2.01 ± 0.26^a^	0.142	0.006
TC level (mg/dl)	223.87 ± 17.67^a^	127.45 ± 31.35^b^	131.30 ± 10.33^b^	20.67	0.013
CAT activity (U/ml)	0.36 ± 0.00	0.29 ± 0.05	0.35 ± 0.03	0.042	0.492
SOD activity (U/ml)	15.76 ± 1.07	11.08 ± 1.22	11.33 ± 1.49	1.537	0.165
NO level (nmol/ml)	21.84 ± 4.99	23.15 ± 2.09	18.06 ± 0.74	3.68	0.632
TAC (mM/L)	0.47 ± 0.07	0.53 ± 0.12	0.40 ± 0.00	0.086	0.577
LPO level (mmol/ml)	4.08 ± 0.23^b^	7.82 ± 0.51^a^	7.92 ± 0.48^a^	0.284	0.001

*TC * Total cholesterol, *CAT * Catalase, *SOD * Superoxide dismutase, *NO * Nitric oxide, *TAC * total antioxidant capacity, *LPO * lipid peroxides

Results are expressed as the mean ± SE of 6 Japanese male quails per group

^a,b^ indicate a significant difference at *p* < 0.05 (One-way ANOVA followed by Duncan’s multiple range test)

## Histological findings

**Fig. 1:**
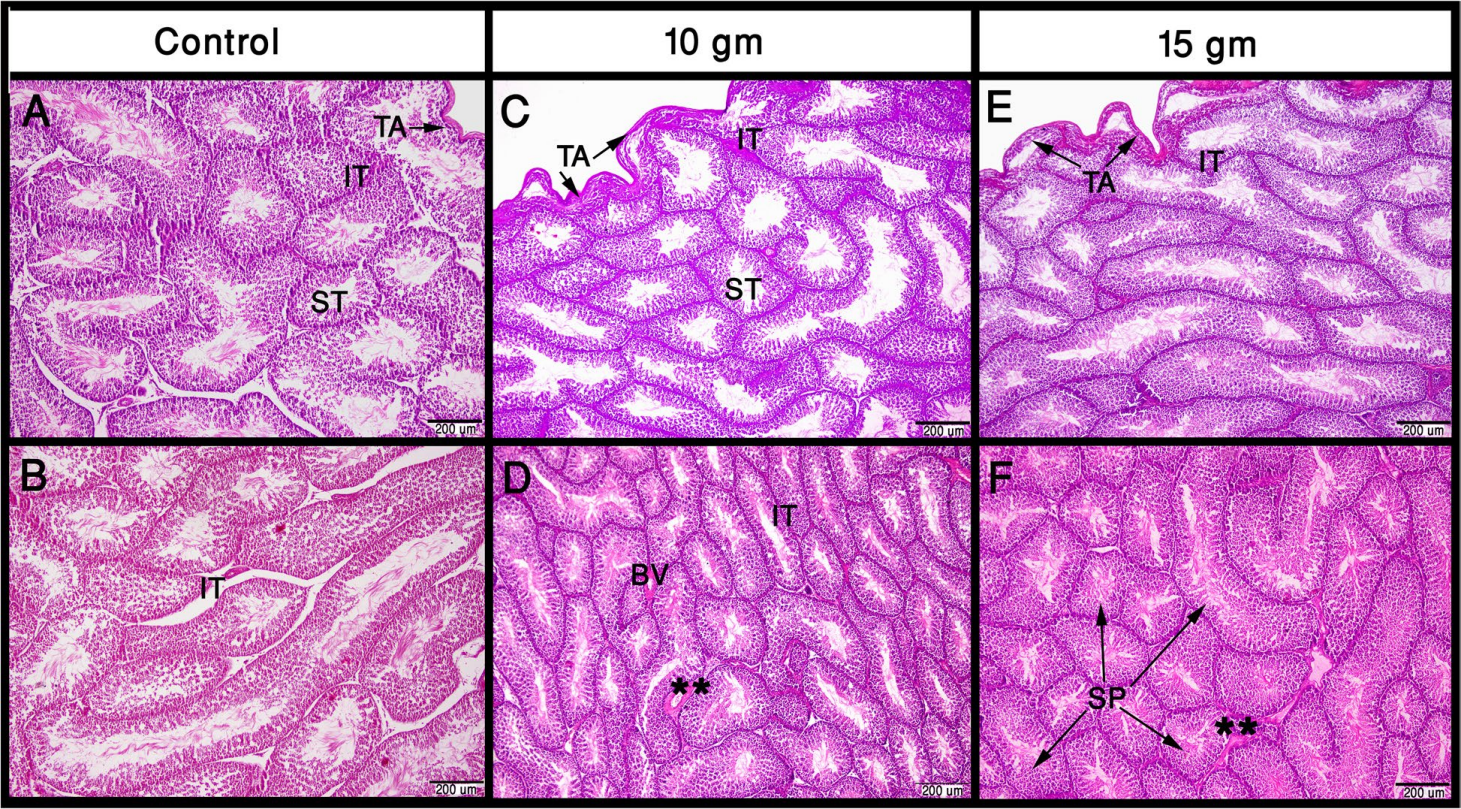
The morphological structure of the quail testes in the control and after feeding on Ginger roots. The testis capsule or tunica albuginea (TA) and interstitial tissue (IT) appear thin in the birds fed on the balanced ration (in the left panel). The seminiferous tubules (ST) in those birds have some spaces in between and a wide lumen. After supplementation with 10 and 15 g of Ginger roots to the ration, the TA became thicker, the IT became more condensed and obvious, and the ST have a narrower lumen because of the thicker spermatogenic epithelium SP. Note the blood vessels (BV) in the Ginger-supplemented groups

The quail testes parenchyma was formed of convoluted seminiferous tubules and interstitial cells. The seminiferous tubules are lined by a stratified epithelium of spermatogenic cells and Sertoli cells (Fig. 1). Noticeably, we found that birds fed on 15 and 30 gm of Ginger roots had well-vascularized interstitial tissue when compared to the control group (Fig. 1).

**Fig. 2:**
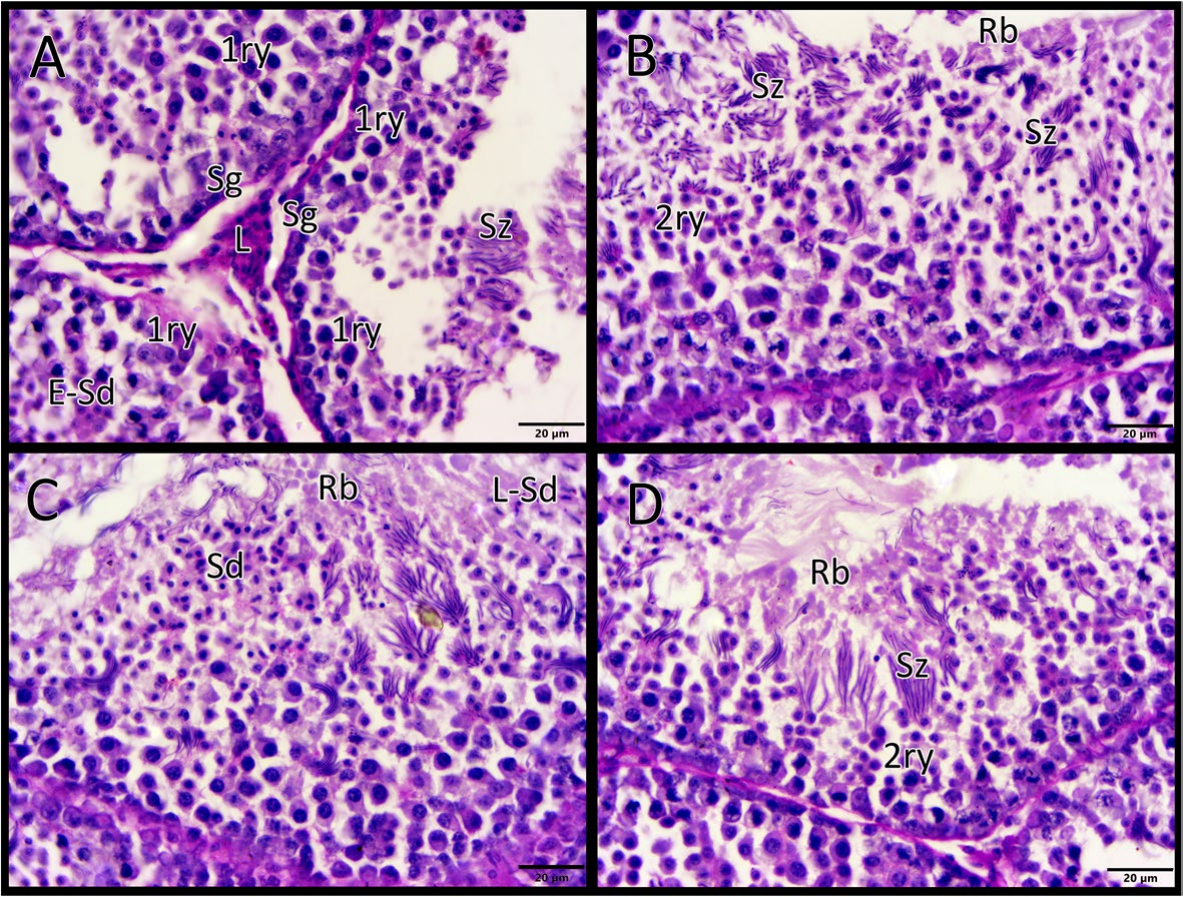
The spermatogenic epithelium after supplementation with Ginger roots. The spermatogenesis has been activated with an increased height of the spermatogenic epithelium. Different stages of spermatogenesis appeared, starting with spermatogonia (Sg), primary spermatocyte (1ry), secondary spermatocyte (2ry), early spermatid (E-Sd), spermatid (Sd), late spermatid (L-Sd) and the spermatozoa (Sz). Note the abundant spermatozoa and residual body (Rb)

The spermatogenic epithelium includes all the stages of spermatogenesis (Fig. 2). First, the mothers of spermatogenic cells, the spermatogonia, are small and round cells with dark nuclei. They lie adjacent to the basement membrane. These cells undergo continuous mitotic divisions and produce the second element, primary spermatocytes. They are larger cells whose nuclei often show distinct chromatin. The primary spermatocytes undergo the first meiotic division, giving rise to the third element, the secondary spermatocytes, which are smaller than the primary spermatocytes. The second meiotic division that occurs shortly gives rise to the fourth element, the haploid spermatids. The early spermatids are rounded cells with pale nuclei that occur in clusters. The late spermatids have condensed chromatin to become elliptical and lie toward the lumen of the seminiferous tubule. Finally, the late spermatids become elongated and transform into the last element, the characteristic sperm of birds. Many cytoplasmic residual bodies detached from the developing spermatids and are found in the lumen of the seminiferous tubule.

**Fig. 3:**
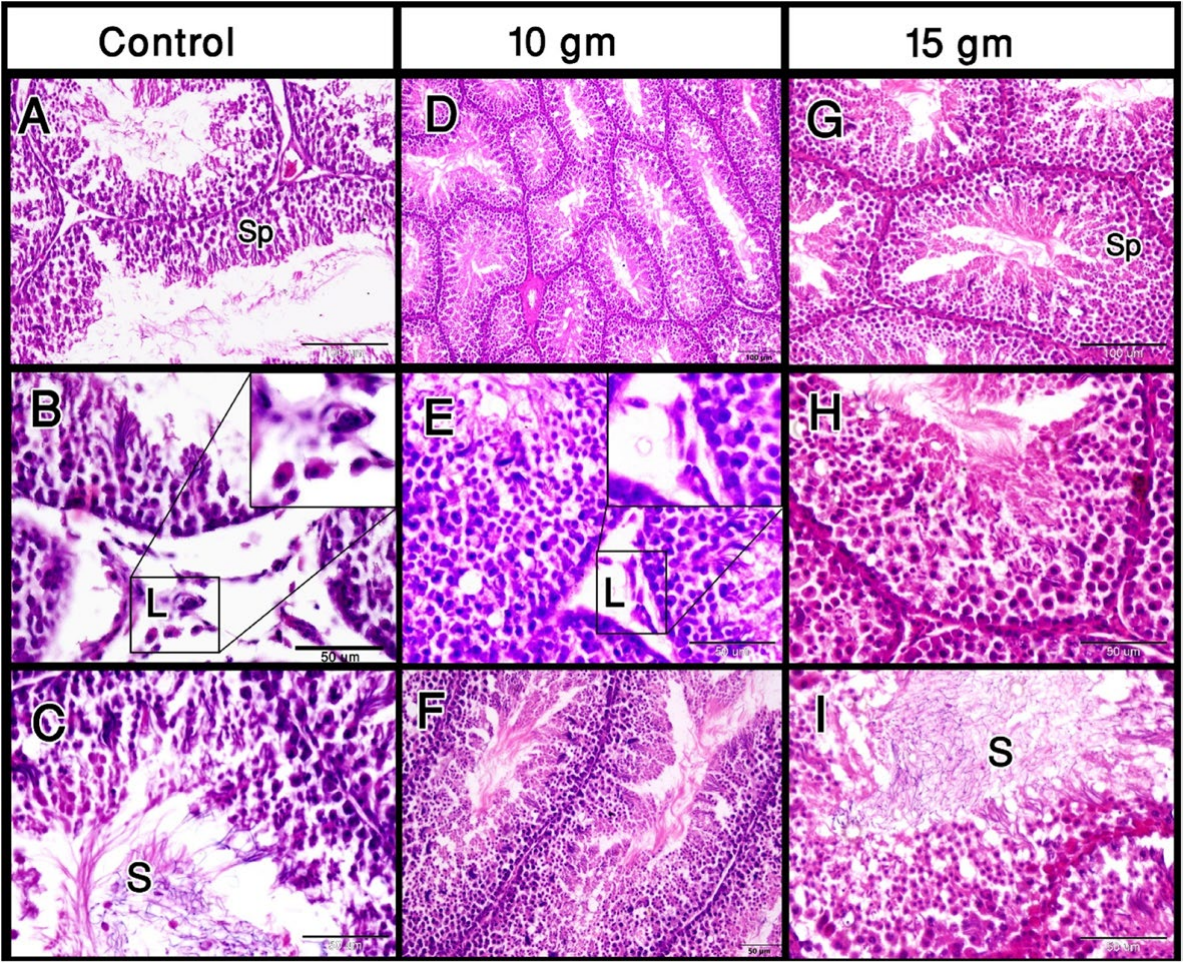
A comparison between the control and Ginger-supplemented groups. The spermatogenic epithelium (SP) became higher and more crowded in both doses of Ginger roots. The Leydig cells (L) are scattered in the wide interstitial tissue in the control. After administration of the Ginger roots, they became closer to each other in the narrower interstitial tissue. Note the more crowded sperms (S) in the lumen of the seminiferous tubules after Ginger supplementation

To determine the effect of oral administration of Ginger roots on the testicular histomorphometric aspects, the diameter of the seminiferous tubules and the height of the spermatogenic epithelium were measured in the Ginger-fed groups in comparison with the control one as indicators of the testicular activity. Surprisingly, the diameter of the seminiferous tubules decreased significantly after the lower dose of Ginger roots, however, it increased significantly after the higher supplementation of Ginger roots. It was obvious that the spermatogenic cell lines/rows in Japanese quails fed on the two doses of Ginger roots were greater than those in the control one. This was determined by the significant increase in the height of the epithelium following supplementation with both doses of Ginger roots (Table 5). Simultaneously, lots of spermatozoa were observed in the lumen. The interstitial tissue is compacted into a thick connective tissue (Fig. 3). The number of Leydig cells increased, however insignificantly, following supplementation with both doses of Ginger roots (Table 5 & Fig. 3).

**Table 5:** Effect of dietary supplementation of Ginger roots to Japanese male quails on the testicular histomorphometric parameters at 10 weeks of age

Group Parameter	Control	Ginger (10 g/Kg feed)	Ginger (15 g/Kg feed)	Pooled SEM	*P* value
Diameter of seminiferous tubule	232.56 ± 2.89^b^	219.14 ± 3.17^c^	254.40 ± 5.02^a^	3.67	0.000
Height of the germinal epithelium	53.28 ± 0.98^b^	57.46 ± 1.55^b^	62.78 ± 2.20^a^	1.75	0.003
Number of Leydig cells	8.83 ± 0.68	8.83 ± 0.60	9.50 ± 0.56	0.56	0.132

Results are expressed as the mean ± SE of 5 Japanese male quails per group

^a,b,c^ Means in the same row with different superscripts are significantly different at *p* < 0.05 (One-way ANOVA followed by Duncan’s multiple range test)

## Discussion

In this study, we aimed to evaluate the male fertility, plasma redox parameters, and histomorphometric features of the testes of quails after the dietary inclusion of Ginger roots. The enhancement in the semen properties (P = 0.000 in all semen traits, and *P* = 0.004 in foam weight) following supplementation with Ginger roots matched previous reports in Japanese quails and broiler breeders [8, 9]. This response is secondary to the marked increase in testosterone output, foam production, and cloacal gland size according to our findings. Ginger feeding augmented testosterone secretion which boosts spermatogenesis and supports the blood-testis barrier by providing nutrients and growth factors to the developing germ cells [39]. It is well established that the cloacal foam contains lactate that is responsible for energizing the quail spermatozoa-driven sperm motility and metabolism [40]. From another perspective, some phytochemical constituents in Ginger roots such as manganese have a direct beneficial effect on the motility and viability of spermatozoa [18]. Given that sperm is abundant in polyunsaturated fatty acids making it is highly vulnerable to peroxidative injury under free radicals' attack [40]. The success of Ginger roots in elevating the proportions of motile, viable, and fertile sperms might be due to the ability of phenolic phytochemicals in Ginger to stabilize the membranes and genetic material of the sperms [41].

A close inspection of our findings revealed a dose-dependent increase (*P* = 0.000) in CAREA and CVOL in parallel with the increase in CFP and SP holes/GD (*P* = 0.000) in Ginger-supplemented quails. This outcome indicates a strong relationship between the cloacal gland size on one side and the rate of foam production and fertilization potential on the other side. Testosterone plays a key role in determining the dimension and secretory capacity of the cloacal gland [29]. The increase in sperm holes per germinal disc in our observations is a direct indication of successful penetration as the foam is a fundamental prerequisite for sperm transport into the female oviduct [42]. This outcome is consistent with the suggestion that foam is a medium for sperm journey along the oviduct [43]. It has been reported that the cloacal gland size, as one of the secondary sexual characteristics, affects the success of natural copulation, e.g. the males with larger cloacal gland areas had more successful attempts than males with small cloacal gland areas [42]. This indicates that female preference for males with larger cloacal glands is most probably due to the high testosterone concentration and mating activity [44]. On the level of the semen traits, males with large glands can produce more semen with less abnormal spermatozoa [45].

The obvious increase in testosterone levels (*P* = 0.006) following Ginger supplementation is in the same line with the up-to-date research [7], which is attributed to an up-regulated pituitary–gonadal axis, increased level of testicular cholesterol, increased testicular weight, and recycling testosterone receptors [14]. Raised testosterone level is linked with acceleration in lipid peroxidation cascade [46] through both androgen-receptor dependent and independent pathways [47, 48] due to the inactivation of mitochondrial enzymes making the mitochondria more susceptible to redox instability [49]. Testosterone also elevated metabolic rates resulting in reactive oxygen species overloading [50]. The hypocholesterolemic action (*P* = 0.013) of Ginger roots in this investigation could be a secondary sign of an increase in the supply of the cholesterol precursor to the steroidogenic pathway in Leydig cells to accelerate testosterone biosynthesis, and hence, increased cholesterol clearance [51]. The hypocholesterolemic action of Ginger roots in the present experiment could be also similar to previous studies which had stated different actions of Ginger such as higher excretion of fecal cholesterol [52], interference with cholesterol absorption [53], inhibition of HMG-CoA reductase, and activation of low-density lipoprotein-receptors [54].

The induction of lipid peroxidation cascade (*P* = 0.001) by Ginger roots is contradictory to the mainstream literature [8, 55]. This outcome reflects an excessive generation of reactive oxidants which triggers the initiation and propagation of lipid peroxidation chain reactions [56]. Nevertheless, evidence had been amassed from the scholarly articles indicating the diversity in the response patterns to supplementation of phytochemical constituents of Ginger regarding the differences in the studied doses and experimental models. For instance, zerumbone enhances the radiosensitivity of colorectal cancer by depletion of reduced glutathione and acts as a cytotoxic agent by promoting reactive oxidants production [57, 58]. Shogaol triggers lipid production cascade in laryngeal cancer cells [59].

TAC is regarded to be the most appropriate measure for estimating the oxidative/reductive potency of a biological specimen taking into consideration the cumulative synergistic action of overall antioxidants present in the sample [60]. In contrast, the estimation of individual antioxidants may give a confusing picture because antioxidants perform their actions through chain-breaking reactions [61]. The shift in redox balance towards the pro-oxidant side, as evident from increased LPO following intake of Ginger roots, could evoke up-regulation of endogenous antioxidant defenses mediated by activation of redox-sensitive transcription factors and its downstream signaling pathways [62]. The absence of significant change in TAC (*P* = 0.577) in the current study signifies the ability of the antioxidant defensive mechanism to overcome reactive oxygen species and restores redox homeostasis.

The testes' histomorphology provided confirmation for the aforementioned findings. By administering 15 g of Ginger roots per kg of feed, we were able to demonstrate a significant increase in the diameter of ST and height of the spermatogenic series. This finding is consistent with that of Shanoon and his colleagues [63] and may be due to an increase in the bioavailability of androgen receptors, an improvement in the cellular replicating and repair machinery [10], and a blocking of the apoptotic cascades [11]. This finding might possibly reflect a rise in spermatogonia and Sertoli cells in seminiferous tubules [64]. This research also shows that testosterone has the ability to increase Sertoli cell proliferation via follicle-stimulating hormone [65], which in turn provides components required for the proliferation and differentiation of germ cells [66]. The increased transcript level of proliferating cell nuclear antigen in the testis might be another contributory factor in the hyperplasia of germinal epithelium [10]. According to Al-Shathly and his collaborators [10], another contributing reason to the hyperplasia of germinal epithelium may be the elevated transcript level of proliferating cell nuclear antigen in the testis. In Ginger groups, there was a considerable increase in the number of Leydig cells that release testosterone. Birds fed 15 and 30 gm of Ginger roots had well-vascularized interstitial tissue, but in the control groups, the blood vessels were tiny and barely detectable. Raised blood flow and testosterone levels are accelerated and increased by larger blood vessels, which also improve spermatogenesis in the testis and ultimately increase fertility [67]. Active phytochemical ingredients in Ginger roots could be valuable in creating a suitable hormonal/redox condition for epithelial cell multiplication. For instance, 6-paradol increased the number of somatic and germ cells and the diameter and thickness of ST in streptozotocin-induced diabetic rats [68]. 6-Gingerol counteracted the reactive oxidants-mediated lipid peroxidation, and sexual hormonal aberrations induced by carbendazim in rats to maintain a typical testicular histo-architecture [17]. The diameter, lumen, and epithelial lining of ST restored following intraperitoneal injection of manganese in formalin-challenged mice owing to its ability to scavenge free radicals and behave as a chain-breaking antioxidant [69]. Therefore, the current research suggests Ginger roots have androgenic and antioxidant properties in quail testis.

## Conclusion

Dietary supplementation of Japanese male quails with the two selected doses of Ginger roots succeeded in enhancing spermatogenesis, semen quality, fertility, hatchability, and testicular histomorphometric characteristics. Generally, the most effective dose in this regard is the highest one (15 g/kg feed). These outcomes probably are due to enhancement in the testosterone secretion and foam production by Ginger feeding. The findings of this study encourage the dietary inclusion of Ginger roots in the ration of male Japanese quails to increase their fertility, and hence, reduce their number. This strategy avoids male fighting and decreases the cost of maintaining a large number of males. We recommend further studies to widen the dosing regimen to estimate its optimal effect on the fertility potential.

## Data Availability

The datasets during the current study available from the corresponding author on reasonable request.

## Abbreviations

1ry: Primary spermatocyte
2ry: Secondary spermatocyte
ANOVA: Analysis of variance
BV: Blood vessels
C/P: Calorie/protein
CAREA: Cloacal gland area
CAT: Catalase
CFP: Cloacal gland foam production
CP: Crude protein
CVOL: Cloacal gland volume
E-Sd: Early spermatid
H&E: Hematoxylin and eosin
H_2_O_2_: Hydrogen peroxide
HMG-CoA: β-Hydroxy β-methylglutaryl-CoA
IT: Interstitial tissue
L: Leydig cells
LPO: Lipid peroxides
L-Sd: Late spermatid
ME: Metabolizable energy
NaCl: Sodium chloride
NO: Nitric oxide
Rb: Residual body
rpm: Revolutions per minute
S: Crowded sperms
SAS: Statistical analysis systems
Sd: Spermatid
SE: Standard error
Sg: Spermatogonia
SOD: Superoxide dismutase
SP: Spermatogenic epithelium
SP holes/GD: Sperm penetration holes per germinal disc
ST: Seminiferous tubules
Sz: Spermatozoa
TA: Tunica albuginea
TAC: Total antioxidant capacity
TC: Total cholesterol
